# The Fibro-Immune Landscape Across Organs: A Single-Cell Comparative Study of Human Fibrotic Diseases

**DOI:** 10.3390/ijms27042017

**Published:** 2026-02-20

**Authors:** Guofei Deng, Yusheng Luo, Xiaorong Lin, Yuzhi Zhang, Yuqing Lin, Yuxi Pan, Yueheng Ruan, Xiaocong Mo, Shuo Fang

**Affiliations:** 1 Scientific Research Center, Department of Oncology, The Seventh Affiliated Hospital of Sun Yat-sen University, Shenzhen 518107, China; denggf6@mail2.sysu.edu.cn (G.D.); luoysh8@mail2.sysu.edu.cn (Y.L.); linxr29@mail2.sysu.edu.cn (X.L.); zhangyzh76@mail2.sysu.edu.cn (Y.Z.); linyq98@mail2.sysu.edu.cn (Y.L.); panyx53@126.com (Y.P.); 2 Guangdong Provincial Key Laboratory of Digestive Cancer Research, The Seventh Affiliated Hospital of Sun Yat-sen University, Shenzhen 518107, China; 3 School of Medicine, Sun Yat-sen University, Shenzhen 518107, China; ruanyh8@mail2.sysu.edu.cn

**Keywords:** fibrosis, single-cell RNA sequencing, stromal–immune crosstalk, organ specificity, anti-fibrotic therapies

## Abstract

Fibrosis is a hallmark of the tumor microenvironment in many solid cancers, driving tumor progression, immune evasion, and treatment resistance; however, the molecular and cellular mechanisms underlying fibrogenesis—particularly stromal–immune crosstalk across organs—remain incompletely understood, compounded by organ-specific heterogeneity and a lack of reliable immune-related biomarkers. To address this, we performed an integrative single-cell RNA sequencing (scRNA-seq) analysis of fibrotic tissues from four major organs—liver, lung, heart, and kidney—alongside non-fibrotic controls, applying unsupervised clustering, trajectory inference, cell–cell communication modeling, and gene set variation analysis (GSVA) to map the fibro-immune landscape. Our analysis revealed both conserved and organ-specific features: fibroblasts were the dominant extracellular matrix (ECM)-producing cells in liver and lung, whereas endothelial-derived stromal populations prevailed in heart and kidney. Immune profiling uncovered distinct infiltration patterns—macrophages displayed organ-specific polarization states; T cells were enriched for tissue-resident subsets in lung and mucosal-associated invariant T (MAIT) cells in liver; and B cells exhibited marked heterogeneity, including a pathogenic interferon-responsive subset prominent in pulmonary fibrosis. GSVA further identified divergent signaling programs across organs and lineages, including TGF-β/TNF-α in the heart, NOTCH/mTOR in the kidney, glycolysis/ROS in the lung, and KRAS/interferon pathways in the liver. Cell–cell communication analysis highlighted robust crosstalk between macrophages, T/B cells, and stromal cells mediated by collagen, laminin, and CXCL signaling axes. Together, this cross-organ atlas delineates a highly heterogeneous fibro-immune ecosystem in human fibrotic diseases, revealing shared mechanisms alongside organ-specific regulatory networks, with immediate translational implications for precision anti-fibrotic therapy, immunomodulatory drug repurposing, and the development of context-specific biomarkers for clinical stratification and therapeutic monitoring.

## 1. Introduction

Fibrosis is a common pathological endpoint of many chronic diseases, capable of affecting nearly every major organ, including the liver, lungs, kidneys, and heart. It is characterized by the excessive deposition of extracellular matrix (ECM) components and aberrant tissue remodeling that progressively erode organ structure and function [1,2]. Globally, fibrotic diseases account for an estimated 35% of all deaths [3], imposing a substantial and growing burden on public health worldwide. Despite significant progress in understanding its molecular mechanisms, effective therapies capable of halting or reversing disease progression remain elusive [4]. Consequently, fibrosis remains a chronic, end-stage condition that poses one of the most formidable challenges in modern medicine. To overcome this challenge, a deeper understanding of its fundamental pathogenesis is required.

Fundamentally, fibrosis results from a dysregulation of the intricate processes governing injury, inflammation, and tissue repair [5]. Under physiological conditions, acute tissue injury triggers a self-limited inflammatory response and regenerative repair, which collectively restore tissue homeostasis. However, upon repetitive or persistent damage, inflammatory signals fail to resolve, driving the tissue into a state of chronic stress characterized by continuously activated immune–stromal interactions [6]. This process drives fibroblasts to acquire a persistently activated phenotype and differentiate into contractile myofibroblasts, which produce excessive collagen and other ECM components [7]. Simultaneously, impaired matrix degradation coupled with progressive tissue stiffening creates a self-reinforcing feedback loop that sustains fibrogenesis [8]. Central signaling networks—including transforming growth factor-β (TGF-β) [9], WNT, Notch, and Hedgehog pathways [10]—coordinate inflammatory, metabolic, and mechanical cues to regulate fibroblast activation and ECM turnover. Collectively, fibrosis can be viewed as a process driven by inflammation, executed by stromal activation, and orchestrated through immune regulation. Notably, fibrotic remodeling shares mechanistic parallels with tumor stroma, including aberrant fibroblast activation, excessive extracellular matrix deposition, and immunosuppressive microenvironment formation. These overlapping features underscore a bidirectional crosstalk in which fibrosis can foster tumorigenesis and tumors often co-opt fibrotic pathways to support growth and immune evasion. Understanding fibrosis thus holds dual significance for chronic disease and cancer biology, offering synergistic opportunities for therapeutic intervention across both contexts.

Despite this intricate understanding of fibrotic pathogenesis, pharmacological strategies against fibrosis have thus far achieved limited clinical success. The antifibrotic agents pirfenidone and nintedanib, which primarily suppress fibroblast activation and ECM synthesis, are approved for idiopathic pulmonary fibrosis [11] and show modest benefits in other disorders. Critically, however, these treatments fail to reverse established fibrotic lesions [12], underscoring that the condition is not merely a consequence of aberrant stromal repair but a multicellular, dynamic process shaped by complex interactions within the fibrotic niche. Recent advances in single-cell technologies have highlighted the pivotal role of the immune microenvironment in regulating both fibrotic progression and potential regression [6]. Monocyte-derived macrophages, T-cell subsets, and their cytokine networks modulate fibroblast activation, ECM metabolism, and tissue homeostasis, collectively determining whether a damaged organ heals or scars. Nevertheless, it remains unclear whether immune–stromal interactions operate through conserved regulatory frameworks across organs or follow organ-specific molecular logic—an unresolved question that hinders an integrated understanding of fibrogenesis.

The limited efficacy of current therapies underscores several fundamental knowledge gaps that persist in the field. While fibrosis presents conserved histopathological hallmarks across organs, it arises from distinct cellular lineages, transcriptional programs, and microenvironmental contexts. Conventional single-organ studies, while invaluable, are inherently limited in their ability to capture the full spectrum of cross-organ mechanistic diversity. Moreover, animal models differ markedly from human disease in immune composition, metabolism, and remodeling dynamics, which limits translational applicability [6]. Compounding these challenges, reliable biomarkers for detecting early immune imbalance or microenvironmental disturbance are still lacking, hindering early diagnosis and timely intervention. Therefore, addressing these gaps necessitates a cross-organ, high-resolution framework to systematically uncover the shared and divergent regulatory mechanisms that drive fibrotic evolution.

The rapid development of single-cell transcriptomic technologies has opened new avenues to decode the cellular ecosystems of fibrotic diseases. By resolving cellular heterogeneity, lineage trajectories, and intercellular communication at unprecedented resolution [13], these approaches provide profound insights into the spatial and functional complexity of tissue remodeling. In this study, we established a comprehensive pan-organ single-cell atlas of fibrosis encompassing the lung, liver, kidney and heart [14,15,16,17]. Through integrative cross-organ analyses, we delineate the transcriptional landscapes and regulatory circuits governing fibroblast evolution and immune microenvironment remodeling. Our goal is to identify conserved and organ-specific drivers of fibrogenesis, define immune-related biomarkers and therapeutic targets, and ultimately provide a conceptual foundation for the development of novel immune modulatory strategies to improve patient outcomes.

## 2. Results

### 2.1. Cross-Organ Integration Reveals Distinct Cellular Compositions and Microenvironmental Heterogeneity in Fibrosis

**Figure 1:**
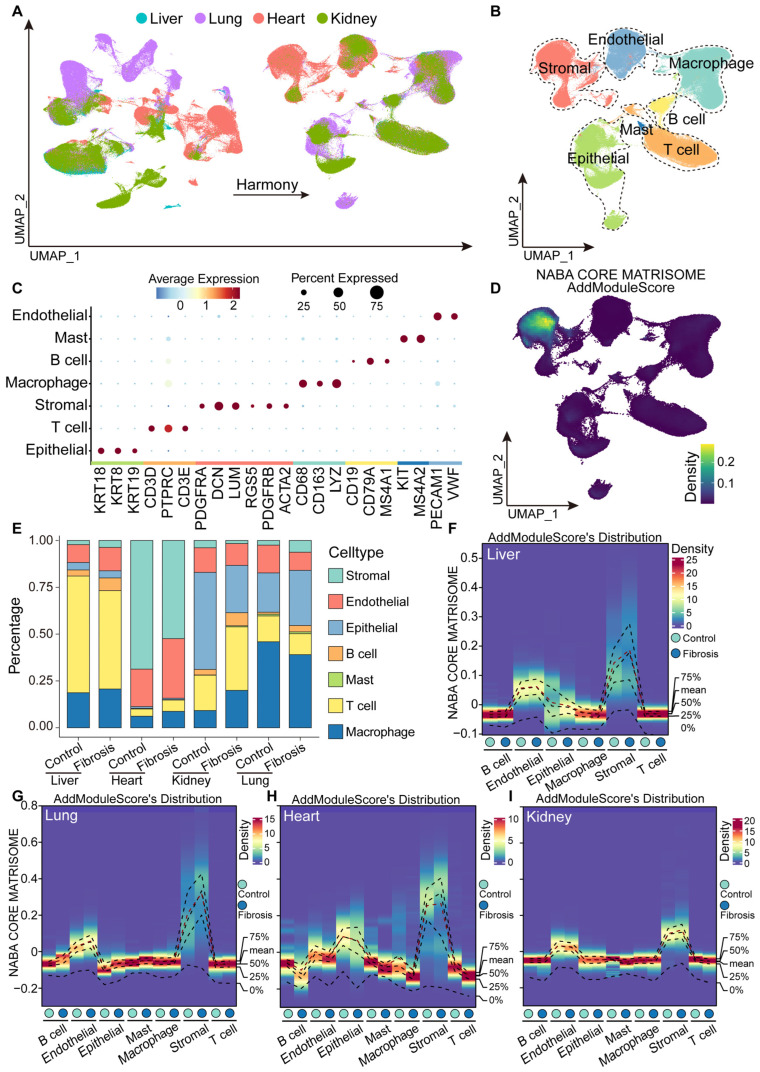
Integrating single-cell data from fibrotic diseases across different organs. (**A**) The single-cell sequencing sources from four organs are subjected to quality control and filtering using Harmony Algorithms. (**B**) After harmony integration, these cells were clustered into seven cell types. (**C**) Markers of different cell types. (**D**) Perform ECM enrichment scoring on cell types using the AddModuleScore algorithm. (**E**) Cell type proportions across organs and disease states. (**F**–**I**) ECM enrichment scores across fibrotic organs. The red dashed line represents the connection of the quartiles of ECM enrichment scores in cell subpopulations across different organs.

To characterize the microenvironmental heterogeneity of fibrosis across different organs, we integrated single-cell RNA-sequencing datasets derived from fibrotic liver, heart, lung, and kidney tissues, together with corresponding normal controls, totaling 75 samples. After quality control, 297,337 cells were retained for downstream analysis. Batch effects originating from different studies were mitigated using the Harmony algorithm (Figure 1A). Following integration, the cells segregated into seven major clusters, which were annotated based on established marker genes: epithelial cells (KRT18, KRT8, KRT19), T cells (CD3D, PTPRC, CD3E), stromal cells (PDGFRA, DCN, LUM, RGS5, PDGFRB, ACTA2), macrophage (CD38, CD163, LYZ), B cells (CD19, CD79A, MS4A1), mast cells (KIT, MS4A2), and endothelial cells (PECAM1, VWF) (Figure 1B,C). Functional enrichment analysis revealed that extracellular matrix (ECM) synthesis-related pathways were predominantly enriched in stromal cells, underscoring their central role in fibrogenesis (Figure 1D). Notably, the cellular composition varied substantially across organs: stromal cells were markedly increased in fibrotic liver and lung tissues, whereas endothelial cells and T cells were relatively enriched in the heart and kidney (Figure 1E). Despite these differences in relative abundance, ECM-producing signatures in stromal cells were consistently upregulated across all organs, indicating pronounced microenvironmental heterogeneity in organ-specific fibrosis (Figure 1F–I).

### 2.2. Subclustering Identifies Stromal Cell Diversity and Organ-Specific Fibrogenic Responses

**Figure 2:**
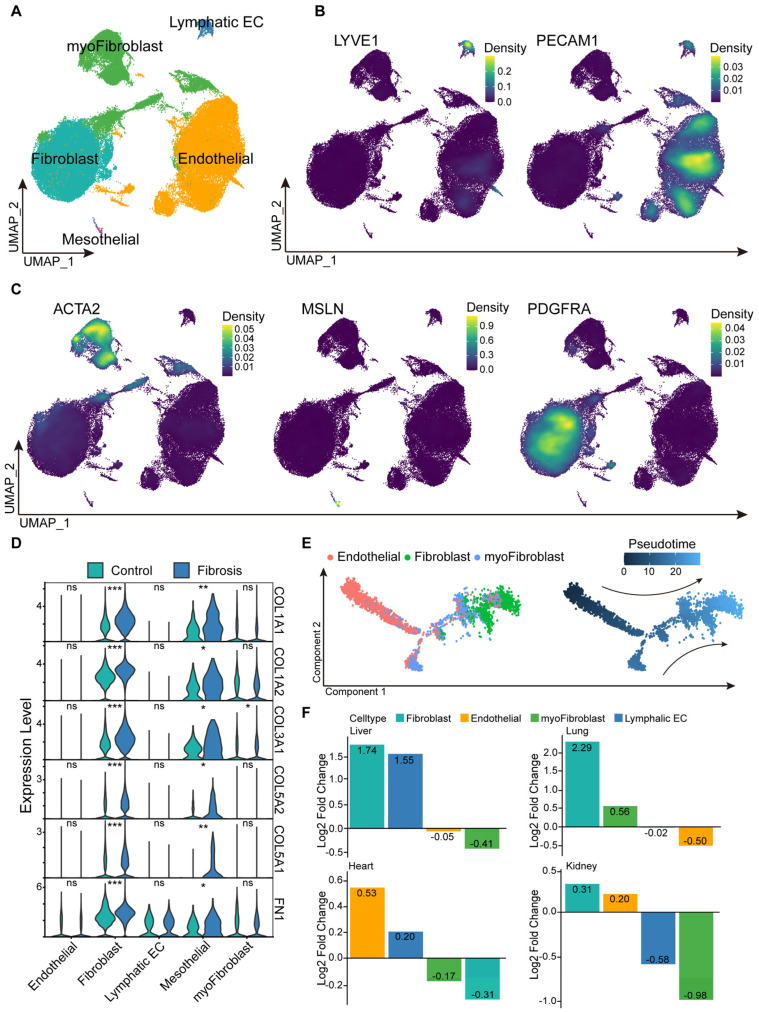
Characterization of Stromal Cell Subsets. (**A**) Subsetting of stromal cells for re-clustering and annotation. (**B**,**C**) Canonical markers for stromal cell annotation. (**D**) Differential expression of collagen genes in stromal cell subsets across fibrotic and healthy tissues. (**E**) Pseudotime analysis of stromal cells. The arrows represent the differentiation trend from fibroblasts or endothelial cells to myofibroblasts, as calculated by the pseudo-time algorithm. (**F**) Proportions of stromal cells across different organs in fibrotic disease models. Statistical significance levels were assigned as *** *p* ≤ 0.001; ** *p* ≤ 0.01; * *p* ≤ 0.05; ns, *p* > 0.05.

Given that stromal cells represented the principal drivers of fibrosis, we performed subclustering to delineate their heterogeneity (Figure 2A). Based on lineage-specific marker gene expression, stromal cells were classified into five major populations (Figure 2B,C). Mesothelial cells were excluded from subsequent analyses due to their low abundance and limited statistical robustness. ECM-related genes were predominantly expressed in fibroblasts and were further upregulated during fibrosis, whereas myofibroblasts exhibited relatively lower expression of these ECM synthetic programs. These findings suggest that fibroblasts serve as the primary ECM-producing population, while myofibroblasts contribute mainly to ECM remodeling and contractile functions (Figure 2D). Pseudotime trajectory analysis further indicated that myofibroblasts largely arose from fibroblasts and endothelial cells through transdifferentiation during fibrogenesis (Figure 2E). We also observed marked organ-specific differences in stromal cell composition (Figure 2F). Fibroblasts were significantly enriched in fibrotic liver and lung tissues, whereas endothelial-derived stromal populations were more prominent in the heart and kidney. These results together suggest that fibroblasts constitute the predominant effector cells in hepatic and pulmonary fibrosis, whereas endothelial-derived stromal cells may play a major role in cardiac and renal fibrogenic responses.

### 2.3. Cell–Cell Communication Analysis Links Three Major Immune Cell Types to Stromal-Driven Fibrosis

**Figure 3:**
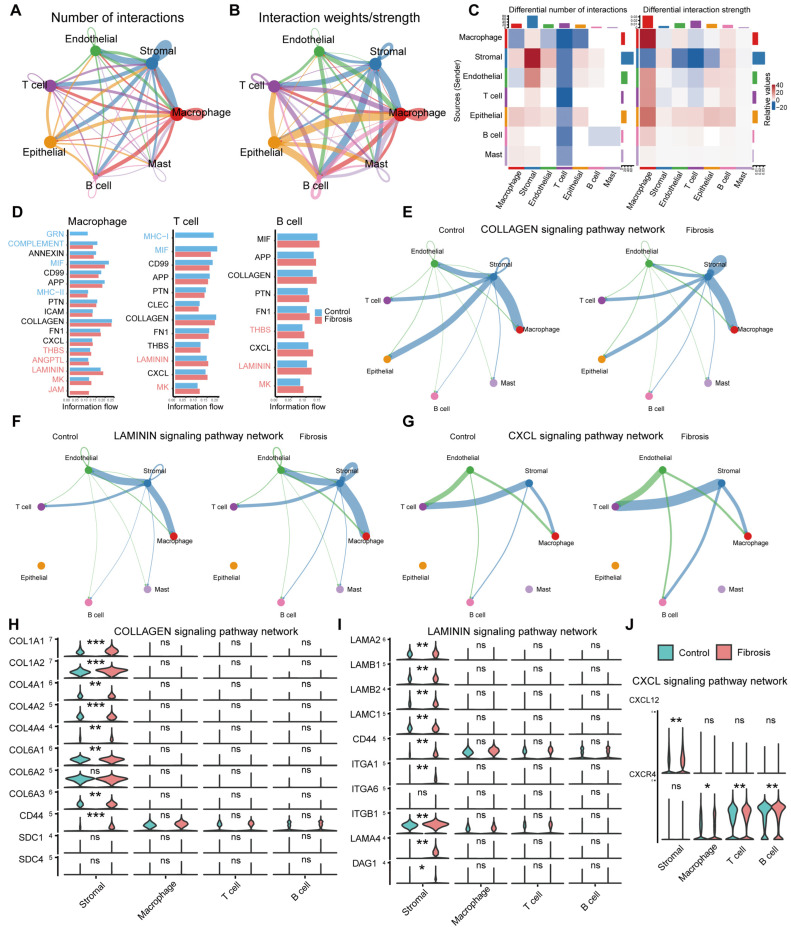
Stromal–immune cell interaction networks in fibrotic diseases. (**A**,**B**) Number and weight of CellChat interactions among seven cell types. (**C**) Heatmap of the number and weight of signaling outputs among seven cell types. (**D**) Activated signaling pathways in three major immune cell types between healthy and disease conditions. (**E**) COLLAGEN signaling pathway network. (**F**) LAMININ signaling pathway network. (**G**) CXCL signaling pathway network. (**H**–**J**) Violin plots of the expression of corresponding signaling pathway ligand–receptor pairs. Statistical significance levels were assigned as *** *p* ≤ 0.001; ** *p* ≤ 0.01; * *p* ≤ 0.05; ns, *p* > 0.05.

To delineate stromal cell interactions in fibrosis, we performed cell–cell communication analysis. The quantity and strength of interactions among the seven major cell types are summarized in Figure 3A,B. Integration with the signaling heatmap (Figure 3C) revealed that macrophages, T cells, and B cells were most strongly associated with stromal cells, prompting our focused analysis on these populations. Comparison of activated pathways between fibrotic and healthy samples revealed significant dysregulation of ECM-related (e.g., COLLAGEN, LAMININ) and inflammation-related (e.g., CXCL) pathways across multiple immune cells (Figure 3D). Having established these key pathways, we constructed detailed signaling networks (Figure 3E–G) to visualize the intensity and weight of cellular crosstalk, which underscored the central role of these three immune cell types in fibrogenic signaling. To further delineate the distinct functions of these cells, we analyzed receptor–ligand pairs for the aforementioned pathways (Figure 3H–J). This analysis identified macrophages as the primary effector cells in ECM-related signaling, while T cells and B cells predominantly mediated inflammatory pathways.

### 2.4. Cross-Organ Macrophage Differentiation Trajectories During Fibrosis

**Figure 4:**
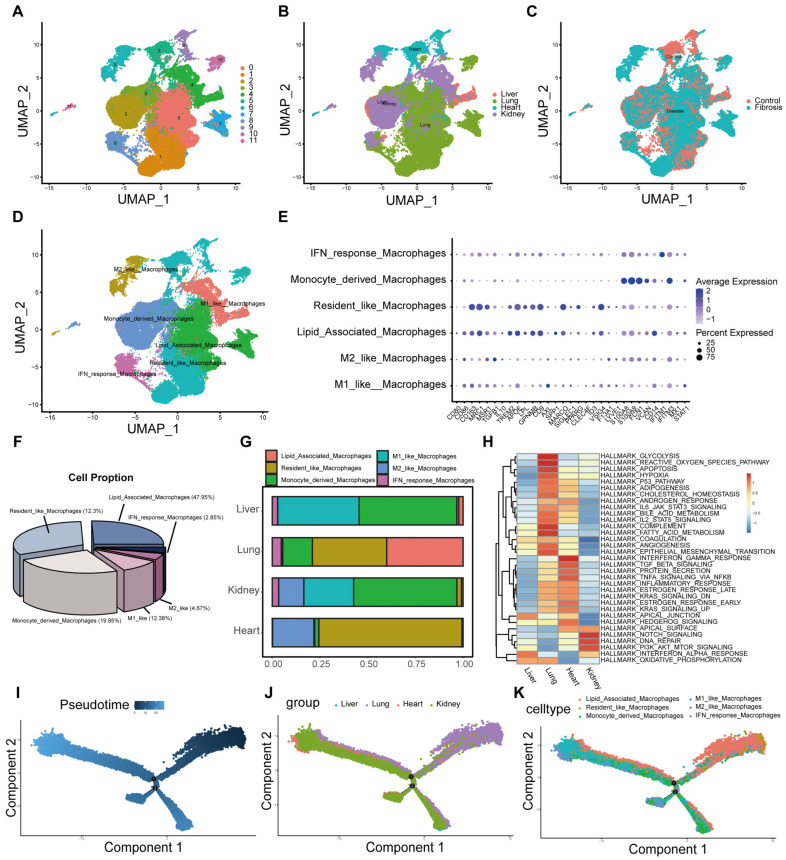
Heterogeneity of Macrophages in Fibrosis Models. (**A**–**C**) Subsetting of Macrophages for Re-clustering. (**D**) Macrophages were annotated into six distinct subtypes. (**E**) Classical macrophage markers used for annotation. (**F**) Proportions of the six macrophage subpopulations. (**G**) Proportions of macrophage subpopulations across different organs. (**H**) Heatmap of GSVA pathway enrichment analysis across different organs. (**I**–**K**) Pseudotime analysis of macrophage subpopulations. The numbers in the figure indicate the two differentiation branches of macrophages.

Macrophages are pivotal drivers of fibrotic diseases. To delineate their heterogeneity and differentiation paths across organs, we performed secondary clustering on macrophage subpopulations, identifying 12 distinct clusters (Figure 4A). We observed striking heterogeneity in macrophage origins across organs and disease states (Figure 4B,C). Based on lineage-specific markers, we annotated these clusters as M1 (CD80, CD86), M2 (CD163, MRC1, MSR1, TGFB1, IL10), lipid-associated (TREM2, APOE, LPL, GPNMB, CD9, AXL, SPP1), resident (MARCO, SIGLEC1, PPARG, CLEC4F, ID3, VSIG4, F13A1, LYVE1), monocyte-derived (S100A8, S100A9, FCN1, VCAN, CD14), and interferon-responsive (IFITM1, IFITM3, MX1, STAT1) macrophages (Figure 4D,E). Beyond common monocyte-derived and resident macrophages, fibrotic samples were notably enriched (47.95%) in lipid-associated macrophages (LAMs) (Figure 4F). Infiltrating macrophage composition varied considerably by organ: monocyte-derived M1 macrophages predominated in the liver, resident and LAMs were prominent in the lungs, M2 macrophages were notably abundant in the kidneys, and the heart was enriched for resident macrophages (Figure 4G). GSVA revealed that these distinct infiltration backgrounds were associated with organ-specific pathway activation: interferon response in the liver; reactive oxygen species and glycolysis in the lungs; TGF-β and TNF-α pathways in the heart; and NOTCH and mTOR signaling in the kidneys (Figure 4H). Finally, pseudotime trajectory analysis revealed two principal differentiation paths in fibrosis: one primarily derived from monocytes, and another originating from resident macrophages and LAMs, both undergoing M1/M2 polarization as the disease progressed, thereby contributing to fibrosis (Figure 4I–K).

### 2.5. Dynamic T Cell Subsets in Fibrosis

**Figure 5:**
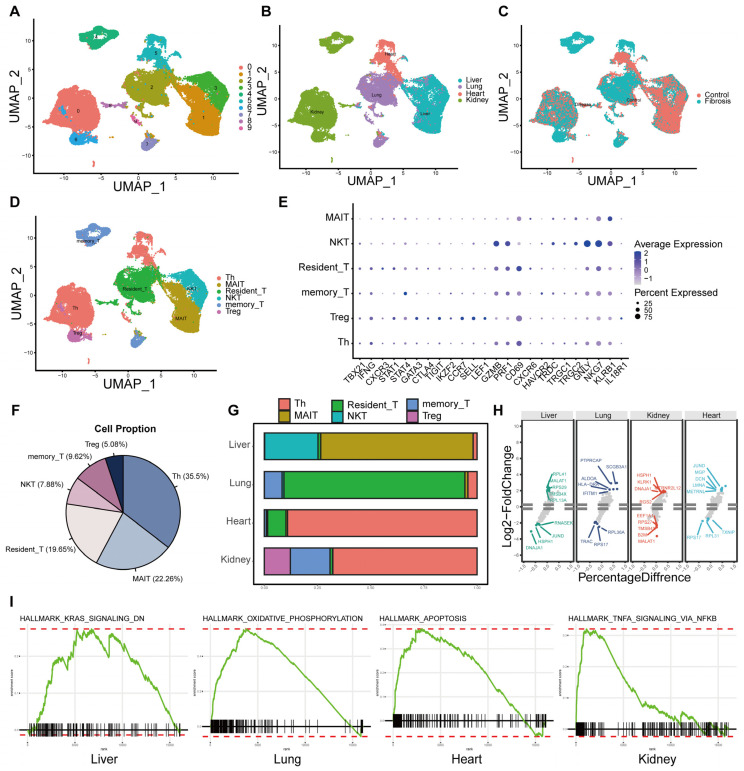
Dynamic changes in T cell subsets in fibrotic diseases. (**A**–**C**) Subsetting of T cells for re-clustering analysis. (**D**) Annotate T cells into six distinct subpopulations. (**E**) Classical T cell markers used for annotation. (**F**) Proportions of the six T cell subpopulations in fibrosis. (**G**) Distribution of T cell subsets among various organs. (**H**) Volcano plot of differentially expressed genes across different organs. (**I**) GSEA analysis results across different organs.

T cells are also pivotal drivers of fibrotic pathogenesis. To systematically characterize their heterogeneity, we performed secondary clustering on T cell subpopulations, identifying 10 distinct clusters (Figure 5A) that exhibited substantial variation in tissue origin and disease state (Figure 5B,C). Using canonical markers, we annotated six major subsets: Helper T cells (TBX21, IFNG, CXCR3, STAT1, STAT4), Regulatory T cells (GATA3, CTLA4, TIGIT, IKZF2), Memory T cells (CCR7, SELL, LEF1, GZMB, PRF1, IFNG), Tissue-resident memory T cells (CD69, CXCR6, HAVCR2), NKT cells (TRDC, TRGC1, TRGC2, GNLY, NKG7), and MAIT cells (KLRB1, TRAV1, IL18R1, CXCR6) (Figure 5D,E). Among these, helper T cells, MAIT cells, and tissue-resident T cells constituted the predominant populations in fibrotic samples (Figure 5F). We observed striking organ-specific infiltration patterns: MAIT and NKT cells were enriched in the liver; tissue-resident T cells dominated in the lung; helper T cells were most abundant in the heart; and regulatory T cells were notably present in the kidney (Figure 5G). We next identified differentially expressed genes in T cells across organs (Figure 5H) and performed GSEA. This revealed T cell-activated signaling pathways distinct from those in macrophages. KRAS signaling was upregulated in liver T cells; oxidative phosphorylation was enriched in lung T cells; autophagy-related pathways were prominent in heart T cells; and TNF-α signaling via NF-κB was dominant in kidney T cells. These findings underscore that distinct immune cell types engage in non-overlapping, organ-specific signaling programs during fibrosis.

### 2.6. B Cell Immunogenicity in Fibrosis

**Figure 6:**
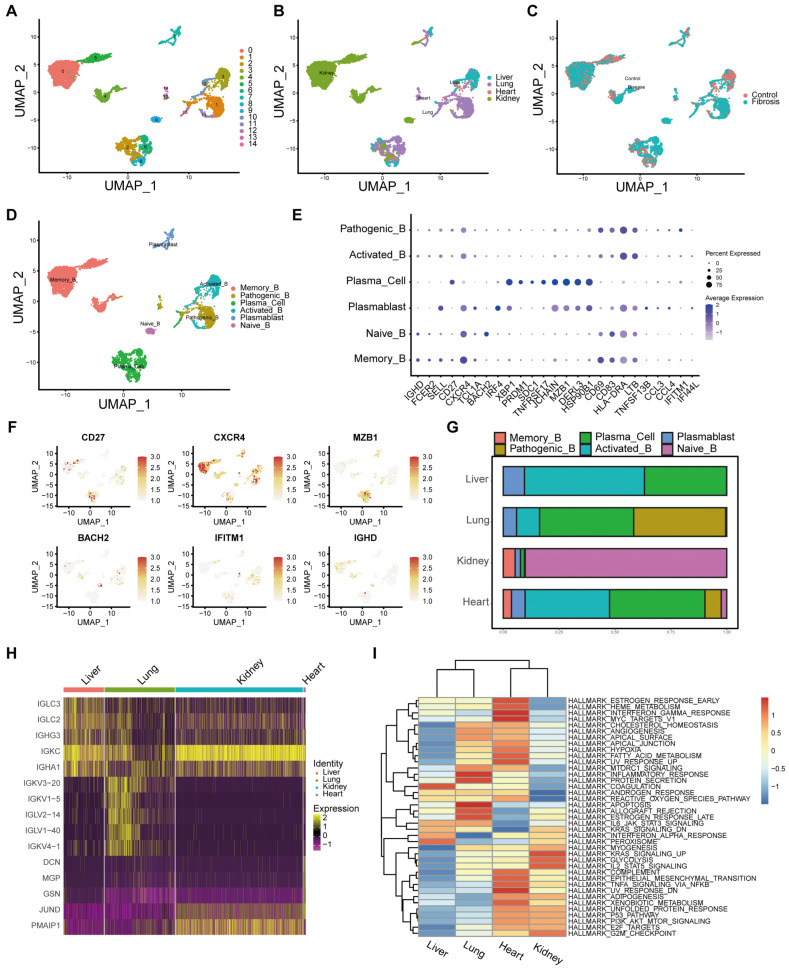
Characterization of B Cell Subsets. (**A**–**C**) Subsetting of B cells for re-clustering. (**D**) Annotation of B cells into six subsets. (**E**) Classical B cell markers used for annotation. (**F**) Genes highly expressed in different B cell subpopulations. (**G**) Proportions of the six B cell subpopulations across different organs. (**H**) Heatmap of highly variable genes across different organs. (**I**) Heatmap of GSVA pathway enrichment analysis across different organs.

B cell infiltration was observed across fibrotic diseases, implicating their potential pro-fibrotic role. To dissect B cell heterogeneity, we performed secondary dimensionality reduction and clustering, which resolved 15 distinct clusters (Figure 6A) with marked variation in organ origin and disease status (Figure 6B,C). Using canonical marker genes, we annotated six major B cell subsets: naïve B cells (IGHD, FCER2, SELL), memory B cells (CD27, CXCR4, TCL1A, BACH2), plasmablasts (IRF4, XBP1, PRDM1, SDC1, TNFRSF17, IGJ), plasma cells (JCHAIN, MZB1, DERL3, XBP1, PRDM1, SDC1, IGLL5, HSP90B1), activated B cells (CD69, CD83, HLA-DRA), and pathogenic B cells (LTB, TNFSF13B, CCL3, CCL4, IFITM1, IFI44L) (Figure 6D,E). Figure 6F illustrates the distribution of highly variable genes across these subsets. Quantification of subset infiltration revealed distinct organ-specific patterns: plasma cells dominated the activated B cell pool in the liver; interferon-responsive pathogenic B cells were most prevalent in the lung; the kidney was enriched for naïve B cells; and the heart exhibited a profile similar to the liver, with prominent plasma cell and memory B cell infiltration (Figure 6G). Differential gene expression analysis further highlighted organ-specific fibrogenic mechanisms. A heatmap of B cell transcriptomes showed not only enrichment of immunoglobulin-related genes, but also organ-wise activation of transcription factors and pro-apoptotic genes—most notably in kidney and heart samples, suggesting distinct pathogenic processes relative to the liver and lung (Figure 6H). GSVA further demonstrated that B cells engaged in organ-specific signaling programs: in the liver, interferon-alpha and KRAS signaling were predominant, aligning with patterns observed in macrophages and T cells; lung B cells were enriched for inflammatory and apoptotic pathways; whereas B cells in the heart and kidney exhibited broad pathway activation, indicative of heightened immunogenicity in these organs (Figure 6I). Collectively, these findings reveal the complex and organ-specific regulatory landscape of B cells in fibrotic pathogenesis.

### 2.7. Validated by Immunohistochemistry and qPCR

**Figure 7:**
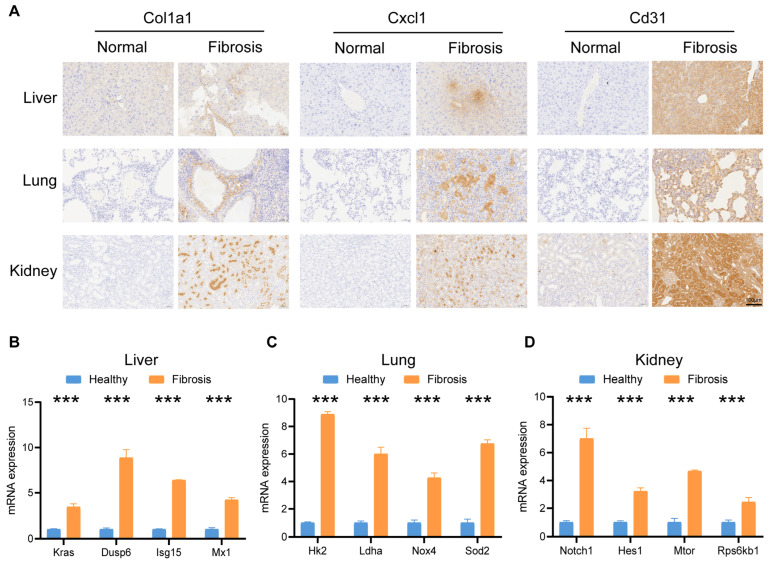
Validated by immunohistochemistry (IHC) and quantitative PCR (qPCR). (**A**) Tissues from mouse models of liver fibrosis, pulmonary fibrosis, and renal fibrosis were subjected to immunohistochemistry. (**B**–**D**) qPCR was used to evaluate pathways potentially activated in fibrotic diseases across different organs. Statistical significance levels were assigned as *** *p* ≤ 0.001.

To further validate the aforementioned findings obtained through single-cell sequencing, we utilized previously established animal models of fibrosis from our research center to acquire tissue samples from mouse models of liver fibrosis, pulmonary fibrosis, and renal fibrosis. We then employed antibodies against Col1a1, Cxcl1, and Cd31 to assess their expression patterns across these organ-specific fibrosis models. The results showed that Col1a1 was expressed to varying degrees in all three fibrosis models—liver, lung, and kidney—consistent with our earlier observation that stromal cells across different fibrotic organs commonly exhibit upregulated ECM production. Cxcl1, a classic inflammatory marker, also demonstrated increased expression in fibrotic lesions compared to healthy controls, suggesting that inflammatory responses represent a core pathway in fibrogenesis. Additionally, Cd31 staining for endothelial cells revealed that endothelial activation was particularly prominent in the renal fibrosis model (Figure 7A).

For elucidating differences in activated signaling pathways among fibrotic processes in distinct organs, we performed qPCR to examine the activity of the KRAS pathway, oxidative phosphorylation-related pathways, and the Notch pathway across multiple organs. The qPCR results aligned well with our prior single-cell sequencing analyses: the KRAS pathway was significantly upregulated in liver fibrosis (Figure 7B), pulmonary fibrosis predominantly involved oxidative phosphorylation-related pathways (Figure 7C), and renal fibrosis was primarily characterized by Notch pathway activation—highlighting the critical role of immune regulation in the pathogenesis of organ-specific fibrosis (Figure 7D).

## 3. Discussion

In this study, we integrated single-cell transcriptomic datasets from fibrotic tissues of the liver, lung, kidney, and heart to generate a cross-organ immune–stromal atlas of human fibrosis. This analysis allowed us to dissect both the conserved and organ-specific components of immune remodeling during fibrotic disease. Across all four organs, stromal cells showed consistently elevated ECM-related programs and a shared trajectory toward myofibroblast activation (Figure 1F–I and Figure 2E,F), supporting the central role of fibroblasts described in previous studies [18]. A set of immune–stromal communication pathways, dominated by COLLAGEN, LAMININ, and CXCL signaling, was also observed across organs (Figure 3E–G), aligning with recent models that highlight immune-driven stromal activation as a fundamental process in fibrosis [6,19]. Despite these shared features, organ-specific immune responses were evident, particularly in the liver, which displayed pronounced innate-like immune activation characterized by expansion of M1-like and monocyte-derived macrophages and enrichment of MAIT and NKT cells (Figure 4G and Figure 5G). This profile differs from the metabolic stress-associated immune activation observed in the lung, the Th-dominant inflammatory pattern in the kidney, and the resident immune-associated stress signatures in the heart. These findings indicate that fibrosis reflects a combination of conserved stromal activation and organ-dependent immune remodeling, rather than being driven by a uniform immune mechanism across tissues.

Comparison across organs further revealed common immune alterations as well as distinct organ-specific signatures. All four tissues demonstrated enhanced stromal activation and ECM upregulation (Figure 2D–F and Figure 7A), accompanied by a consistent shift in macrophages toward an M1-like phenotype and recruitment of monocyte-derived macrophages (Figure 4I–K). Regulatory T-cell reduction or impaired functional signatures (Figure 5E–G) also appeared broadly across organs and are consistent with the reported link between Treg imbalance and chronic tissue injury [20]. In addition to these shared processes, each organ displayed distinct immune characteristics shaped by its local microenvironment. In hepatic fibrosis, in addition to the commonly observed expansion of M1-like macrophages and monocyte-derived macrophages (Figure 4G), lipid-associated-macrophages (LAMs) also participate to a certain extent in the regulation of the inflammation–repair balance, suggesting that the endogenous lipid microenvironment of the liver may shape additional immune regulatory nodes. The enrichment of MAIT and NKT cells further highlights the liver’s unique innate-like immune landscape. In contrast, lung fibrosis was marked by increased oxidative phosphorylation and glycolysis pathways (Figure 5I and Figure 6I), consistent with the lung’s dependence on oxygen metabolism [21,22]. Kidney fibrosis exhibited increased plasma-cell infiltration and activation of immunoglobulin-associated and TNF-α–NF-κB pathways (Figure 5I and Figure 7B–D), suggesting roles for chronic inflammation and potential autoantibody involvement, similar to immune complex-mediated injury [23]. Cardiac fibrosis was characterized by activation of apoptosis-related programs in tissue-resident T cells (Figure 5I), indicating local immune dysregulation and cell stress responses [24]. These observations illustrate that, although fibrosis shares a common immune–stromal axis, its progression is shaped by organ-specific immune ecosystems, with the liver displaying the most extensive immune remodeling. The conserved immune–stromal network we identified mirrors the immunosuppressive microenvironment in advanced tumors, underscoring fibrosis not only as a feature of chronic organ damage but also as a critical contributor to cancer-associated stromal dysfunction.

Among the original studies we previously included, GSE135893, GSE168933, and GSE198204 primarily focused on the roles of stromal cells—including mesenchymal cells, endothelial cells, and fibroblasts—in individual organs during sclerotic diseases, largely overlooking the critical contribution of the immune microenvironment to fibrotic disease progression. In contrast, GSE211785 characterized the fibrotic microenvironment (FME) in the kidney but lacked high-resolution, cross-organ human data capable of capturing the heterogeneity of immune responses across tissues. These limitations have long kept fibrosis research staying at the level of common mechanisms, and the understanding of organ-specific immune-driven mechanisms remains insufficient. Based on the above foundation, our study, for the first time, constructed a human multi-organ fibrosis immune atlas at the single-cell level across the liver, lung, kidney, and heart, revealing shared and divergent immune trajectories during the fibrotic process. Particularly in the liver, we observed a unique immune composition and a more complex inflammatory amplification pattern, including prominent expansion of M1-like and monocyte-derived macrophages, as well as enrichment of MAIT/NKT cells, indicating that the liver exhibits the strongest innate-like immune remodeling characteristics among the multi-organ comparison. In addition, we identified a previously overlooked immune subset known as LAMs. Their presence in fibrotic liver tissue suggests that the lipid environment may shape unique nodes of immune activation, expanding the new dimensions of fibrosis immune regulation. More importantly, by systematically integrating the cross-organ remodeling characteristics of macrophage, T cell, and B cell subsets, we revealed a highly conserved and structurally stable immune–stromal cell interaction network within the fibrotic microenvironment, forming a network communication loop across multiple immune and stromal cell types. This multi-level interaction not only persists in all organs but also progressively strengthens with the progression of fibrosis, indicating that it is not a downstream accompanying phenomenon of fibrosis but rather the core driving engine for ECM accumulation and tissue remodeling, which is highly consistent with the immune-driven fibrosis model proposed in recent years [6,19].

The cross-organ immune atlas generated in this study not only delineates organ-specific and shared features of fibrotic immune landscapes but also provides a robust foundation for clinical translation. Organ-specific differences in immune composition reveal promising early diagnostic biomarkers: for instance, the emergence of LAMs in liver fibrosis reflects an early shift in immune–lipid signaling, suggesting that combined metrics of lipid metabolism and immune activity could serve as sensitive indicators of hepatic fibrogenesis. Similarly, endothelial activation and angiogenesis signatures in kidney and heart point to microvascular remodeling as a potential early cue for disease detection. Beyond diagnostics, the conserved immune–stromal network we identified—strikingly reminiscent of the immunosuppressive microenvironment in advanced tumors—unlocks new therapeutic opportunities. Shared pathways across organs, including persistent IFN signaling, stress responses, and dysregulated cell death, highlight core mechanisms amenable to broad-spectrum anti-fibrotic strategies, while recent insights into immunometabolism, apoptosis regulation, and immune–stromal crosstalk [6,25,26] are strongly supported by our single-cell data. Critically, organ-specific immune ecosystems enable precision intervention: in liver fibrosis, targeting LAM polarization or lipid-sensing receptors (e.g., PPARγ, LXR) and harnessing MAIT cell cytotoxicity may restore inflammation–repair balance and counteract stromal immunosuppression; in renal fibrosis, modulating naïve B-cell activity offers a more targeted alternative to global anti-inflammatory approaches; and in cardiac fibrosis, mitigating T-cell-associated apoptosis may be more effective than broad immunosuppression. Moreover, given the metabolic rewiring observed—particularly enhanced glycolysis and oxidative phosphorylation in lung fibrosis—combining metabolic modulators (e.g., metformin, dichloroacetate) with immunotherapies warrants investigation. Most compellingly, the parallels between fibrotic and tumor microenvironments suggest that immune checkpoint inhibitors (e.g., anti-PD-1/PD-L1, anti-CTLA-4), though often ineffective in fibrosis-rich cancers, could regain potency when paired with anti-fibrotic agents (e.g., LOXL2 or FAK inhibitors) or stroma-modulating regimens, thereby dismantling both physical and immunological barriers to enhance drug delivery and T-cell infiltration. Future clinical trials must therefore stratify patients by organ-specific fibro-immune signatures to enable precision targeting and maximize therapeutic efficacy.

Although this study has constructed a systematic immune atlas of cross-organ fibrosis, there are still several limitations. Firstly, the limitation of sample size inevitably affects the universality of immune composition inference, particularly in tissues such as the heart, where the relatively low number of available single cells may result in certain low-abundance immune subpopulations not being adequately captured. Secondly, the inherent nature of single-cell transcriptomics primarily reveals associations and state characteristics rather than causal relationships. While we observed cross-organ common immune–stromal interaction networks that suggest potential functional connections, these inferences still require further validation through co-culture, organoid, or animal models to confirm their true role in the fibrotic process. Furthermore, this study has not incorporated spatial dimension information. Although a schematic of immune–stromal interactions through co-receptor analysis has been constructed, the spatial topological structure of these interactions in real tissues cannot be directly resolved by single-cell data. Future integration of spatial transcriptomics and spatial proteomics will allow more precise mapping of immune-cell localization within specific fibrotic niches in different organs, which will be of great value for understanding their unique fibrotic trajectories.

## 4. Materials and Methods

### 4.1. Data Sources

The single-cell RNA sequencing (scRNA-seq) datasets from human fibrotic tissues were obtained from the Gene Expression Omnibus (GEO) repository (https://www.ncbi.nlm.nih.gov/geo/, accessed on 1 November 2025). To investigate transcriptional alterations across multiple organ-specific fibrotic microenvironments, four publicly available datasets representing pulmonary fibrosis (GSE135893), hepatic fibrosis (GSE168933), cardiac fibrosis (GSE198204), and renal fibrosis (GSE211785) were included in this study [14,15,16,17]. Each dataset contained fibrotic tissues paired with non-fibrotic controls. All samples were downloaded as raw expression matrices or raw sequencing files, ensuring standardized preprocessing across studies for subsequent analyses. Metadata associated with the datasets are summarized in Table S1. Only human-derived data were included, and all datasets originated from previously published studies with ethical approval reported by the original investigators.

### 4.2. Human Fibrotic Conditions Included

The datasets analyzed were derived from human subjects diagnosed with organ-specific fibrotic diseases, including pulmonary fibrosis, hepatic fibrosis, cardiac fibrosis, and renal fibrosis. These conditions represent major clinical forms of chronic tissue remodeling characterized by excessive ECM deposition and progressive loss of organ function. The included samples encompass tissues obtained from patients with Idiopathic fibrosis (IPF), cirrhotic liver of various etiologies, atrial fibrillation associated with fibrosis, and renal fibrosis related to chronic kidney disease. Each dataset also includes corresponding non-fibrotic or healthy control tissues, enabling comparative analysis of transcriptional alterations associated with fibrotic progression.

### 4.3. Data Loading, Quality Control, and Preprocessing

The raw single-cell RNA sequencing data from the included human fibrotic tissue datasets, formatted according to 10× Genomics specifications, were imported into R and processed using the CreateSeuratObject function from the Seurat package (version 4.3.0). To remove low-quality and sparsely detected transcripts, the parameters were configured to exclude genes detected in fewer than five cells (min.cells = 5) and to retain cells expressing at least 300 genes (min.features = 300). For human-derived samples, the proportion of mitochondrial transcripts was calculated using the PercentageFeatureSet function with the pattern “^MT-”. Cells with 200–6000 detected genes (nFeature_RNA) and mitochondrial UMI proportions below 20% were retained, ensuring the exclusion of apoptotic, stressed, or low-quality cells. All filtering procedures were performed using the subset function.

Following quality control, gene expression values were normalized using the NormalizeData function with the “LogNormalize” method and a scale factor of 10,000. Highly variable genes (HVGs) were identified using the FindVariableFeatures function with the “vst” method, selecting the top 2000 HVGs to capture the most informative transcriptional signals. The resulting gene expression matrices were scaled using the ScaleData function, during which mitochondrial transcript proportion (percent.mt) and sequencing depth (nCount_RNA) were regressed out to mitigate their confounding effects. The processed and standardized expression matrix was subsequently used for dimensionality reduction and downstream analyses.

### 4.4. Dimensionality Reduction and Batch Integration

Principal component analysis (PCA) was performed on the scaled highly variable genes (HVGs) using the RunPCA function in Seurat (version 4.3.0) with the number of principal components (npcs) set to 50. Based on elbow plot inspection and the cumulative variance explained, the top 20 principal components (PCs) were selected for subsequent analyses [27]. Because the four included GEO datasets originated from different organs and were generated in independent studies, dataset-specific technical variations were addressed using Harmony (version 0.1.1). Integration was performed using the RunHarmony function with “dataset” specified as the grouping variable (group.by.vars = “dataset”), reduction = “pca”, dims.use = 1:20, and max.iter.harmony = 10. Harmony-corrected embeddings were subsequently used for downstream dimensionality reduction and visualization. The effectiveness of integration was evaluated by comparing UMAP and PCA embeddings before and after Harmony correction using DimPlot [28], ensuring that dataset-related batch effects were mitigated without obscuring biologically meaningful inter-organ transcriptional differences.

### 4.5. Cell Clustering and Visualization

A shared nearest neighbor (SNN) graph was constructed using Harmony-corrected principal component embeddings based on the top 20 dimensions (FindNeighbors, reduction = “harmony”, dims = 1:20). Graph-based clustering was then performed using the Louvain algorithm with a resolution parameter of 0.1 (FindClusters). Cellular heterogeneity was visualized in two dimensions using Uniform Manifold Approximation and Projection (UMAP) via the RunUMAP function, implemented on the Harmony-integrated embedding space (reduction = “harmony”, dims = 1:20, n.neighbors = 30, min.dist = 0.3). The resulting UMAP representations were visualized utilizing DimPlot, with cells colored by cluster identity or sample group as appropriate.

### 4.6. Cell Type Annotation and Subpopulation Analysis

Cluster-specific marker genes were identified using the FindAllMarkers function with stringent filtering criteria (only.pos = TRUE, min.pct = 0.25, logfc.threshold = 0.25, test.use = “wilcox”). Cell type annotation was initially performed by comparing cluster-enriched marker genes with well-established human single-cell reference signatures reported in previous studies and curated marker databases. Annotation accuracy was further confirmed by examining gene expression patterns using FeaturePlot and VlnPlot.

To resolve cellular heterogeneity within major lineages, clusters corresponding to specific cell types were isolated using the subset function. Each subset underwent an independent re-analysis pipeline, including data renormalization, identification of highly variable genes (HVGs), principal component analysis (PCA) using the top 20 principal components, and higher-resolution subclustering (FindClusters, resolution = 0.05). UMAP visualization and marker gene identification were subsequently repeated for each subpopulation. Subclustering analyses were performed independently for each lineage without additional dataset integration to preserve organ-specific transcriptional distinctions. The full list of marker genes used for cell type annotation is provided in Table S2.

### 4.7. Differential Expression and Gene Set Variation Analysis (GSVA)

Differentially expressed genes (DEGs) between fibrotic and corresponding non-fibrotic control tissues were identified for each organ-specific dataset using the FindMarkers function in Seurat. The filtering criteria included a minimum detection fraction of 0.25 (min.pct = 0.25), a minimum log2 fold-change threshold of 0.25 (logfc.threshold = 0.25), and the “MAST” statistical test. Genes with an adjusted *p*-value < 0.05 were considered statistically significant.

To explore pathway-level alterations associated with fibrotic progression, Gene Set Variation Analysis (GSVA) was performed using the normalized expression matrix via the “GSVA” R package (version 1.46.0). Hallmark gene sets from the Molecular Signatures Database (MSigDB) (http://www.gsea-msigdb.org/gsea/index.jsp, accessed on 20 July 2024) were retrieved using the “msigdbr” package (species set to Homo sapiens; category “H: Hallmark gene sets”). GSVA enrichment scores were computed for each cell or sample, enabling the assessment of biological pathway activity across fibrotic and control groups.

### 4.8. Pseudo-Time Trajectory Analysis

Pseudotime trajectory analysis was performed to infer the continuous transitions of cellular states associated with fibrotic progression. Lineage-specific subsets (e.g., fibroblasts, macrophages, endothelial cells) were isolated based on their annotated identities and subjected to trajectory reconstruction using the Monocle package (version 2.26.0). A cell_data_set object was constructed from the normalized expression matrix, and highly variable genes identified within each subset were used as ordering features. Dimensionality reduction was performed using Uniform Manifold Approximation and Projection (UMAP), followed by principal graph learning to generate the trajectory structure. Pseudotime values were computed using the order_cells function, assigning the root state according to clusters enriched for control or less-activated phenotypes. This approach enabled the visualization of dynamic transcriptional transitions and the identification of key molecular changes associated with fibrotic state progression.

### 4.9. Cell–Cell Communication Analysis

Cell–cell communication analysis was performed independently for each organ using the CellChat R package (available at https://github.com/sqjin/CellChat, accessed on 4 August 2024). For each dataset, a CellChat object was created using normalized expression data and cell type annotations. Ligand–receptor interactions were inferred based on the CellChatDB.human database, retaining pairs in which both components were expressed in at least 10% of cells within a given population. Communication probabilities were computed using computeCommunProb and aggregated at the pathway level with computeCommunProbPathway. The overall signaling networks were summarized with aggregateNet and visualized using standard CellChat functions, including circle plots, hierarchy plots, and chord diagrams [29]. All analyses were conducted separately for pulmonary, hepatic, cardiac, and renal tissues to preserve organ-specific signaling characteristics.

### 4.10. Quantitative Real-Time PCR (qPCR)

Total RNA was isolated from snap-frozen fibrotic and age-matched control tissues of mouse liver, lung, heart, and kidney using TRIzol reagent (Invitrogen, CA, USA). One microgram of total RNA was reverse-transcribed into cDNA using the HiScript IV All-in-One Ultra RT SuperMix for qPCR (Vazyme, Shanghai, China) with random primers, according to the manufacturer’s protocol. Negative controls without reverse transcriptase (–RT) were included to verify absence of genomic DNA amplification. Quantitative real-time PCR was performed in duplicate on a CFX96™ Real-Time PCR System (BIO-RAD, CA, USA) using TaqProUniversal SYBR qPCR Master Mix (Vazyme, Shanghai, China). Gene-specific primers (listed in Supplementary Table S4) were designed to span exon–exon junctions based on Mus musculus RefSeq sequences and validated for amplification efficiency (90–110%) and single-peak melting curves.

### 4.11. Immunohistochemistry (IHC)

Tissues from mouse liver, lung and kidney were harvested at the experimental endpoint, fixed in 4% paraformaldehyde (PFA) for 48 h at 4 °C, and subsequently embedded in paraffin after dehydration through a graded ethanol series. Serial sections (4–5 μm thick) were cut using a rotary microtome and mounted on microscope slides. For immunohistochemical staining, sections were deparaffinized in xylene and rehydrated through a descending ethanol gradient to distilled water. Antigen retrieval was performed by heating slides in EDTA buffer (1 mM EDTA, pH 8.0), depending on the primary antibody requirement, using a pressure cooker or microwave for 10–15 min. Endogenous peroxidase activity was blocked with 3% hydrogen peroxide in methanol for 15 min at room temperature, followed by incubation in blocking serum (5% normal goat or donkey serum in PBS) for 1 h to reduce non-specific binding. Primary antibodies were diluted in antibody diluent and applied overnight at 4 °C. The following primary antibodies were used: Col1a1, Cxcl1, Cd31. After washing with PBS, sections were incubated with HRP-conjugated secondary antibodies for 1 h at room temperature. Immunoreactivity was visualized using 3,3′-diaminobenzidine (DAB) chromogen (Vazyme, Shanghai, China), followed by counterstaining with hematoxylin. Slides were dehydrated, cleared in xylene, and coverslipped with permanent mounting medium.

### 4.12. Statistical Analysis

Statistical analyses were conducted using the R software (version 4.1.2) and GraphPad Prism (version 8.3.0). Data quantifications are reported as means ± standard deviation (SD). For comparisons among three or more groups, one-way ANOVA with Dunnett’s multiple comparisons test was used. For comparisons between two groups, a two-tailed Student *t*-test was applied. Statistical significance was assessed using a threshold of *p* < 0.05.

## 5. Conclusions

In conclusion, this study presents a cross-organ single-cell immune atlas that characterizes both conserved and organ-specific immune remodeling processes during fibrosis, and constructs a comprehensive immune–fibrotic interaction landscape. Our work not only uncovers conserved mechanisms driving fibroblast activation and immune response amplification, but also elucidates marked inter-organ disparities in macrophage and T/B cell remodeling, as well as immune–stromal crosstalk. These insights establish a novel conceptual framework for understanding fibrosis heterogeneity. Collectively, our findings advance the understanding of fibrotic immunopathology and lay the groundwork for the development of targeted and personalized immune-based anti-fibrotic therapies in the future.

## Data Availability

All datasets analyzed in this study are publicly available in the NCBI GEO repository (accession numbers: GSE135893, GSE168933, GSE198204 and GSE211785). Links to the datasets are provided in ‘Methods’ and ‘Supplementary File’.

## Supplementary Materials

The following supporting information can be downloaded at: https://www.mdpi.com/article/10.3390/ijms27042017/s1. References [30,31,32,33,34,35,36,37,38,39,40,41,42,43,44,45,46,47,48,49,50,51,52,53,54,55,56,57,58,59,60,61,62,63,64,65,66,67,68,69,70,71,72,73,74,75,76,77,78,79,80,81,82,83,84,85,86,87,88,89,90,91,92] are cited in the Supplementary Materials.
