# Supplementary material for: The Fibro-Immune Landscape Across Organs: A Single-Cell Comparative Study of Human Fibrotic Diseases
Source: Int J Mol Sci. 2026 Feb 20;27(4):2017. doi: 10.3390/ijms27042017 (PMC12940318; doi:10.3390/ijms27042017)
Supplement: Supplementary file 1 [file ijms-27-02017-s001.zip › ijms-4036106-supplementary.pdf]

Supplementary Table S1. The detailed information of the included GEO datasets.

Series	Type	Number of samples	Organism	Number of cells	PMID
GSE135893	Pulmonary fibrosis	31	Homo sapiens	100416	32832598
GSE168933	Hepatic fibrosis	12	Homo sapiens	30140	35074474
GSE198204	Atrial fibrosis	6	Homo sapiens	80620	40626706
GSE211785	Renal fibrosis	26	Homo sapiens	86161	39048792

Supplementary Table S2. QC statistics for each organ-specific dataset.

Series	Raw cells (pre-QC)	Cells retained (post-QC)	Cells removed
GSE135893	114396	100416	13980
GSE168933	38079	30140	7939
GSE198204	82194	80620	1574
GSE211785	338565	86161	252404

Supplementary Table S3. Marker genes applied in cell annotation.

Cell type	Marker	Reference
Epithelial cells	KRT18<br>KRT8	[30] Toivola, D. M.; Ku, N.-O.; Resurreccion, E. Z.; Nelson, D. R.; Wright, T. L.; Omary, M. B. Keratin 8 and 18 Hyperphosphorylation Is a Marker of Progression of Human Liver Disease. *Hepatology.* **2004**, 40, 459–466.
	KRT19	[31] Govaere, O.; Komuta, M.; Berkers, J.; Spee, B.; Janssen, C.; de Luca, F.; Katoonizadeh, A.; Wouters, J.; van Kempen, L. C.; Durnez, A.; Verslype, C.; De Kock, J.; Rogiers, V.; van Grunsven, L. A.; Topal, B.; Pirenne, J.; Vankelecom, H.; Nevens, F.; van den Oord, J.; Pinzani, M.; Roskams, T. Keratin 19: A Key Role Player in the Invasion of Human Hepatocellular Carcinomas. *Gut*. **2014**, 63, 674–685.
T cells	CD3D<br>PTPRC<br>CD3E	[32] Zhong, Y.; Wei, B.; Wang, W.; Chen, J.; Wu, W.; Liang, L.; Huang, X.-R.; Szeto, C.-C.; Yu, X.; Nikolic-Paterson, D. J.; Lan, H.-Y. Single-Cell RNA-Sequencing Identifies Bone Marrow-Derived Progenitor Cells as a Main Source of Extracellular Matrix-Producing Cells across Multiple Organ-Based Fibrotic Diseases. *Int. J. Biol. Sci.* **2024**, 20, 5027–5042.
Stromal cells	PDGFRA<br>DCN<br>LUM	[33] Payen, V. L.; Lavergne, A.; Alevra Sarika, N.; Colonval, M.; Karim, L.; Deckers, M.; Najimi, M.; Coppieters, W.; Charloteaux, B.; Sokal, E. M.; El Taghdouini, A. Single-Cell RNA Sequencing of Human

		Liver Reveals Hepatic Stellate Cell Heterogeneity. *JHEP rep.: innov. hepatol.* **2021**, 3, 100278.
	RGS5<br>PDGFRB<br>ACTA2	[32] Zhong, Y.; Wei, B.; Wang, W.; Chen, J.; Wu, W.; Liang, L.; Huang, X.-R.; Szeto, C.-C.; Yu, X.; Nikolic-Paterson, D. J.; Lan, H.-Y. Single-Cell RNA-Sequencing Identifies Bone Marrow-Derived Progenitor Cells as a Main Source of Extracellular Matrix-Producing Cells across Multiple Organ-Based Fibrotic Diseases. *Int. J. Biol. Sci.* **2024**, 20, 5027–5042.
Macrophage	CD68<br>CD163<br>LYZ	[34] MacParland, S. A.; Liu, J. C.; Ma, X.-Z.; Innes, B. T.; Bartczak, A. M.; Gage, B. K.; Manuel, J.; Khuu, N.; Echeverri, J.; Linares, I.; Gupta, R.; Cheng, M. L.; Liu, L. Y.; Camat, D.; Chung, S. W.; Seliga, R. K.; Shao, Z.; Lee, E.; Ogawa, S.; Ogawa, M.; Wilson, M. D.; Fish, J. E.; Selzner, M.; Ghanekar, A.; Grant, D.; Greig, P.; Sapisochin, G.; Selzner, N.; Winegarden, N.; Adeyi, O.; Keller, G.; Bader, G. D.; McGilvray, I. D. Single Cell RNA Sequencing of Human Liver Reveals Distinct Intrahepatic Macrophage Populations. *Nat. Commun.* **2018**, 9, 4383.
B cells	CD19<br>CD79A<br>MS4A1	[35] Shan, Y.; Qi, D.; Zhang, L.; Wu, L.; Li, W.; Liu, H.; Li, T.; Fu, Z.; Bao, H.; Song, S. Single-Cell RNA-Seq Revealing the Immune Features of Donor Liver during Liver Transplantation. *Front. Immunol.* **2023**, 14, 1096733.
Mast cells	KIT	[36] Andrews, T. S.; Atif, J.; Liu, J. C.; Perciani, C. T.; Ma, X.-Z.; Thoeni, C.; Slyper, M.; Eraslan, G.; Segerstolpe, A.; Manuel, J.; Chung, S.; Winter, E.; Cirlan, I.; Khuu, N.; Fischer, S.; Rozenblatt-Rosen, O.; Regev, A.; McGilvray, I. D.; Bader, G. D.; MacParland, S. A. Single-Cell, Single-Nucleus, and Spatial RNA Sequencing of the Human Liver Identifies Cholangiocyte and Mesenchymal Heterogeneity. *Hepatol. Commun.* **2022**, 6, 821–840.
	MS4A2	[37] Luo, X.; Luo, B.; Fei, L.; Zhang, Q.; Liang, X.; Chen, Y.; Zhou, X. MS4A Superfamily Molecules in Tumors, Alzheimer's and Autoimmune Diseases. *Front. Immunol.* **2024**, 15, 1481494.
Endothelial cells	PECAM1<br>VWF	[38] Zhang, J.; Lu, T.; Lu, S.; Ma, S.; Han, D.; Zhang, K.; Xu, C.; Liu, S.; Gan, L.; Wu, X.; Yang, F.; Wen, W.; Qin, W. Single-Cell Analysis of Multiple Cancer Types

		Reveals Differences in Endothelial Cells between Tumors and Normal Tissues. *Comput. Struct. Biotechnol. J.* **2023**, 21, 665–676.
Lipid associated macrophages	TREM2<br>APOE<br>LPL<br>GPNMB<br>CD9<br>AXL<br>SPP1	[39] Jaitin, D. A.; Adlung, L.; Thaiss, C. A.; Weiner, A.; Li, B.; Descamps, H.; Lundgren, P.; Bleriot, C.; Liu, Z.; Deczkowska, A.; Keren-Shaul, H.; David, E.; Zmora, N.; Eldar, S. M.; Lubezky, N.; Shibolet, O.; Hill, D. A.; Lazar, M. A.; Colonna, M.; Ginhoux, F.; Shapiro, H.; Elinav, E.; Amit, I. Lipid-Associated Macrophages Control Metabolic Homeostasis in a Trem2-Dependent Manner. *Cell*. **2019**, 178, 686-698.e14.
Lung resident-like macrophages	MARCO	[40] Sinjab, A.; Han, G.; Treekitkarnmongkol, W.; Hara, K.; Brennan, P. M.; Dang, M.; Hao, D.; Wang, R.; Dai, E.; Dejima, H.; Zhang, J.; Bogatenkova, E.; Sanchez-Espiridion, B.; Chang, K.; Little, D. R.; Bazzi, S.; Tran, L. M.; Krysan, K.; Behrens, C.; Duose, D. Y.; Parra, E. R.; Raso, M. G.; Solis, L. M.; Fukuoka, J.; Zhang, J.; Sepesi, B.; Cascone, T.; Byers, L. A.; Gibbons, D. L.; Chen, J.; Moghaddam, S. J.; Ostrin, E. J.; Rosen, D.; Heymach, J. V.; Scheet, P.; Dubinett, S. M.; Fujimoto, J.; Wistuba, I. I.; Stevenson, C. S.; Spira, A.; Wang, L.; Kadara, H. Resolving the Spatial and Cellular Architecture of Lung Adenocarcinoma by Multiregion Single-Cell Sequencing. *Cancer Discov*. **2021**, 11, 2506–2523.
	SIGLEC1	[41] Shi, T.; Denney, L.; An, H.; Ho, L.-P.; Zheng, Y. Alveolar and Lung Interstitial Macrophages: Definitions, Functions, and Roles in Lung Fibrosis. *J. Leukocyte Biol.* **2021**, 110, 107–114.
	PPARG	[42] Mannes, P. Z.; Adams, T. S.; Farsijani, S.; Barnes, C. E.; Latoche, J. D.; Day, K. E.; Nedrow, J. R.; Ahangari, F.; Kaminski, N.; Lee, J. S.; Tavakoli, S. Noninvasive Assessment of the Lung Inflammation-Fibrosis Axis by Targeted Imaging of CMKLR1. *Sci. Adv.* **2024**, 10, eadm9817.
Liver resident-like macrophages	CLEC4F	[43] Guillot, A.; Tacke, F. Liver Macrophages: Old Dogmas and New Insights. *Hepatol. Commun*. **2019**, 3, 731–743.
	ID3	[44] Deng, Z.; Loyher, P.-L.; Lazarov, T.; Li, L.; Shen, Z.; Bhinder, B.; Yang, H.; Zhong, Y.; Alberdi, A.; Massague,

	VSIG4	J.; Sun, J. C.; Benezra, R.; Glass, C. K.; Elemento, O.; Iacobuzio-Donahue, C. A.; Geissmann, F. The Nuclear Factor ID3 Endows Macrophages with a Potent Anti-Tumour Activity. *Nature.* **2024**, 626, 864–873.
Kidney resident-like macrophages	F13A1	[45] Wang, Z.; Deng, Q.; Gu, Y.; Li, M.; Chen, Y.; Wang, J.; Zhang, Y.; Zhang, J.; Hu, Q.; Zhang, S.; Chen, W.; Chen, Z.; Li, J.; Wang, X.; Liang, H. Integrated Single-Nucleus Sequencing and Spatial Architecture Analysis Identified Distinct Injured-Proximal Tubular Types in Calculi Rats. *Cell Biosci.* **2023**, 13, 92.
	LYVE1	[46] Lee, H.-W.; Qin, Y.-X.; Kim, Y.-M.; Park, E.-Y.; Hwang, J.-S.; Huo, G.-H.; Yang, C.-W.; Kim, W.-Y.; Kim, J. Expression of Lymphatic Endothelium-Specific Hyaluronan Receptor LYVE-1 in the Developing Mouse Kidney. *Cell Tissue Res.* **2011**, 343, 429–444.
Monocyte-derived macrophages	S100A8<br>S100A9	[40] Sinjab, A.; Han, G.; Treekitkarnmongkol, W.; Hara, K.; Brennan, P. M.; Dang, M.; Hao, D.; Wang, R.; Dai, E.; Dejima, H.; Zhang, J.; Bogatenkova, E.; Sanchez-Espiridion, B.; Chang, K.; Little, D. R.; Bazzi, S.; Tran, L. M.; Krysan, K.; Behrens, C.; Duose, D. Y.; Parra, E. R.; Raso, M. G.; Solis, L. M.; Fukuoka, J.; Zhang, J.; Sepesi, B.; Cascone, T.; Byers, L. A.; Gibbons, D. L.; Chen, J.; Moghaddam, S. J.; Ostrin, E. J.; Rosen, D.; Heymach, J. V.; Scheet, P.; Dubinett, S. M.; Fujimoto, J.; Wistuba, I. I.; Stevenson, C. S.; Spira, A.; Wang, L.; Kadara, H. Resolving the Spatial and Cellular Architecture of Lung Adenocarcinoma by Multiregion Single-Cell Sequencing. *Cancer Discov*. **2021**, 11, 2506–2523.
	FCN1<br>VCAN<br>CD14	[47] Sun, X.-F.; Luo, W.-C.; Huang, S.-Q.; Zheng, Y.-J.; Xiao, L.; Zhang, Z.-W.; Liu, R.-H.; Zhong, Z.-W.; Song, J.-Q.; Nan, K.; Qiu, Z.-X.; Zhong, J.; Miao, C.-H. Immune-Cell Signatures of Persistent Inflammation, Immunosuppression, and Catabolism Syndrome after Sepsis. *Med.* **2025**, 6, 100569.
M1 like macrophages	CD80<br>CD86	[48] Azizi, E.; Carr, A. J.; Plitas, G.; Cornish, A. E.; Konopacki, C.; Prabhakaran, S.; Nainys, J.; Wu, K.; Kiseliovas, V.; Setty, M.; Choi, K.; Fromme, R. M.; Dao, P.; McKenney, P. T.; Wasti, R. C.; Kadaveru, K.; Mazutis, L.; Rudensky, A. Y.; Pe'er, D. Single-Cell Map of Diverse Immune Phenotypes in the Breast Tumor

		Microenvironment. *Cell.* **2018**, 174, 1293-1308.e36.
M2 like macrophages	CD163<br>TGFB1	[49] Murray, P. J.; Wynn, T. A. Protective and Pathogenic Functions of Macrophage Subsets. *Nat. Rev. Immunol.* **2011**, 11, 723–737.
	MRC1<br>MSR1	[50] Zhao, S.-J.; Kong, F.-Q.; Jie, J.; Li, Q.; Liu, H.; Xu, A.-D.; Yang, Y.-Q.; Jiang, B.; Wang, D.-D.; Zhou, Z.-Q.; Tang, P.-Y.; Chen, J.; Wang, Q.; Zhou, Z.; Chen, Q.; Yin, G.-Y.; Zhang, H.-W.; Fan, J. Macrophage MSR1 Promotes BMSC Osteogenic Differentiation and M2-like Polarization by Activating PI3K/AKT/GSK3β/β-Catenin Pathway. *Theranostics.* **2020**, 10, 17–35.
	IL10	[51] Wang, N.; Liang, H.; Zen, K. Molecular Mechanisms That Influence the Macrophage M1-M2 Polarization Balance. *Front. Immunol.* **2014**, 5, 614.
IFN-response macrophages	IFITM1<br>IFITM3	[52] Regino-Zamarripa, N. E.; Ramírez-Martínez, G.; Jiménez-Álvarez, L. A.; Cruz-Lagunas, A.; Gómez-García, I. A.; Ignacio-Cortés, S.; Márquez-García, J. E.; Pacheco-Hernández, L. M.; Ramírez-Noyola, J. A.; Barquera, R.; Mendoza-Milla, C.; Luna-Rivero, C.; Domínguez-Cherit, J. G.; Ramírez-Rangel, R.; Rodríguez-Reyna, T. S.; Hernández-Cárdenas, C. M.; Choreño-Parra, J. A.; León-Ávila, G.; Zúñiga, J. Differential Leukocyte Expression of IFITM1 and IFITM3 in Patients with Severe Pandemic Influenza a(H1N1) and COVID-19. *J. Interferon Cytokine Res.: Off. J. Int. Soc. Interferon Cytokine Res.* **2022**, 42, 431–443.
	MX1	[53] Uccellini, M. B.; García-Sastre, A. ISRE-Reporter Mouse Reveals High Basal and Induced Type I IFN Responses in Inflammatory Monocytes. *Cell Rep.* **2018**, 25, 2784-2796.e3.
	STAT1	[54] Schroder, K.; Spille, M.; Pilz, A.; Lattin, J.; Bode, K. A.; Irvine, K. M.; Burrows, A. D.; Ravasi, T.; Weighardt, H.; Stacey, K. J.; Decker, T.; Hume, D. A.; Dalpke, A. H.; Sweet, M. J. Differential Effects of CpG DNA on IFN-Beta Induction and STAT1 Activation in Murine Macrophages versus Dendritic Cells: Alternatively Activated STAT1 Negatively Regulates TLR Signaling in Macrophages. J. *Immunol.* **2007**, 179, 3495–3503.
$CD4^+$ T helper cells	TBX21	[55] Szabo, S. J.; Kim, S. T.; Costa, G. L.; Zhang, X.; Fathman, C. G.; Glimcher, L. H. A Novel Transcription

		Factor, T-Bet, Directs Th1 Lineage Commitment. *Cell*. **2000**, 100, 655–669.
	IFNG<br>STAT1<br>STAT4	[56] Pawlak, M.; Ho, A. W.; Kuchroo, V. K. Cytokines and Transcription Factors in the Differentiation of CD4+ T Helper Cell Subsets and Induction of Tissue Inflammation and Autoimmunity. *Curr. Opin. Immunol.* **2020**, 67, 57–67.
	CXCR3	[57] Lukyanov, D. K.; Kriukova, V. V.; Ladell, K.; Shagina, I. A.; Staroverov, D. B.; Minasian, B. E.; Fedosova, A. S.; Shelyakin, P.; Suchalko, O. N.; Komkov, A. Y.; Blagodatskikh, K. A.; Miners, K. L.; Britanova, O. V.; Franke, A.; Price, D. A.; Chudakov, D. M. Repertoire-Based Mapping and Time-Tracking of T Helper Cell Subsets in scRNA-Seq. *Front. Immunol.* **2025**, 16, 1536302.
Tregs	GATA3	[58] Wang, Y.; Su, M. A.; Wan, Y. Y. An Essential Role of the Transcription Factor GATA-3 for the Function of Regulatory T Cells. *Immunity*. **2011**, 35, 337–348.
	CTLA4	[59] Domínguez Conde, C.; Xu, C.; Jarvis, L. B.; Rainbow, D. B.; Wells, S. B.; Gomes, T.; Howlett, S. K.; Suchanek, O.; Polanski, K.; King, H. W.; Mamanova, L.; Huang, N.; Szabo, P. A.; Richardson, L.; Bolt, L.; Fasouli, E. S.; Mahbubani, K. T.; Prete, M.; Tuck, L.; Richoz, N.; Tuong, Z. K.; Campos, L.; Mousa, H. S.; Needham, E. J.; Pritchard, S.; Li, T.; Elmentaite, R.; Park, J.; Rahmani, E.; Chen, D.; Menon, D. K.; Bayraktar, O. A.; James, L. K.; Meyer, K. B.; Yosef, N.; Clatworthy, M. R.; Sims, P. A.; Farber, D. L.; Saeb-Parsy, K.; Jones, J. L.; Teichmann, S. A. Cross-Tissue Immune Cell Analysis Reveals Tissue-Specific Features in Humans. *Science*. **2022**, 376, eabl5197.
	TIGIT	[60] Joller, N.; Lozano, E.; Burkett, P. R.; Patel, B.; Xiao, S.; Zhu, C.; Xia, J.; Tan, T. G.; Sefik, E.; Yajnik, V.; Sharpe, A. H.; Quintana, F. J.; Mathis, D.; Benoist, C.; Hafler, D. A.; Kuchroo, V. K. Treg Cells Expressing the Coinhibitory Molecule TIGIT Selectively Inhibit Proinflammatory Th1 and Th17 Cell Responses. *Immunity.* **2014**, 40, 569–581.
	IKZF2	[61] Thornton, A. M.; Shevach, E. M. Helios: Still behind the Clouds. *Immunology.* **2019**, 158, 161–170.

Memory T cells	CCR7<br>SELL	[62] Fazeli, P.; Kalani, M.; Hosseini, M. T Memory Stem Cell Characteristics in Autoimmune Diseases and Their Promising Therapeutic Values. *Front. Immunol.* **2023**, 14, 1204231.
	LEF1	[63] Zhou, X.; Xue, H.-H. Cutting Edge: Generation of Memory Precursors and Functional Memory CD8+ T Cells Depends on T Cell Factor-1 and Lymphoid Enhancer-Binding Factor-1. *J. Immunol.* **2012**, 189, 2722–2726.
	GZMB<br>IFNG	[64] Li, X.; Li, S.; Wang, Y.; Zhou, X.; Wang, F.; Muhammad, I.; Luo, Y.; Sun, Y.; Liu, D.; Wu, B.; Teng, D.; Wang, J.; Zhao, K.; Ling, Q.; Cai, J. Single Cell RNA-Sequencing Delineates CD8+ Tissue Resident Memory T Cells Maintaining Rejection in Liver Transplantation. *Theranostics.* **2024**, 14, 4844–4860.
	PRF1	[65] Knörck, A.; Schäfer, G.; Alansary, D.; Richter, J.; Thurner, L.; Hoth, M.; Schwarz, E. C. Cytotoxic Efficiency of Human CD8+ T Cell Memory Subtypes. *Front. Immunol.* **2022**, 13, 838484.
Resident T cells	CD69<br>CXCR6	[66] Bartsch, L. M.; Damasio, M. P. S.; Subudhi, S.; Drescher, H. K. Tissue-Resident Memory T Cells in the Liver-Unique Characteristics of Local Specialists. *Cells.* **2020**, 9, 2457.
	HAVCR2	[67] Li, H.; Yang, D.; Hao, M.; Liu, H. Differential Expression of HAVCR2 Gene in Pan-Cancer: A Potential Biomarker for Survival and Immunotherapy. *Front. Genet.* **2022**, 13, 972664.
Natural killer T cells	TRDC<br>TRGC1<br>TRGC2	[68] Cerapio, J. P.; Perrier, M.; Pont, F.; Tosolini, M.; Laurent, C.; Bertani, S.; Fournie, J.-J. Single-Cell RNAseq Profiling of Human Γδ T Lymphocytes in Virus-Related Cancers and COVID-19 Disease. *Viruses.* **2021**, 13, 2212.
	GNLY<br>NKG7	[69] Gao, C.; Wang, S.; Xie, X.; Ramadori, P.; Li, X.; Liu, X.; Ding, X.; Liang, J.; Xu, B.; Feng, Y.; Tan, X.; Wang, H.; Zhang, Y.; Zhang, H.; Zhang, T.; Mi, P.; Li, S.; Zhang, C.; Yuan, D.; Heikenwalder, M.; Zhang, P. Single-Cell Profiling of Intrahepatic Immune Cells Reveals an Expansion of Tissue-Resident Cytotoxic CD4+ T Lymphocyte Subset Associated with Pathogenesis of Alcoholic-Associated Liver Diseases. *Cell. Mol.*

		Gastroenterol. Hepatol. **2025**, 19, 101411.
Mucosal-associated invariant T cells	KLRB1	[70] Fergusson, J. R.; Hühn, M. H.; Swadling, L.; Walker, L. J.; Kurioka, A.; Llibre, A.; Bertoletti, A.; Holländer, G.; Newell, E. W.; Davis, M. M.; Sverremark-Ekström, E.; Powrie, F.; Capone, S.; Folgori, A.; Barnes, E.; Willberg, C. B.; Ussher, J. E.; Klenerman, P. CD161(Int)CD8+ T Cells: A Novel Population of Highly Functional, Memory CD8+ T Cells Enriched within the Gut. *Mucosal Immunol.* **2016**, 9, 401–413.
	TRAV1<br>IL18R1<br>CXCR6	[71] Fang, Y.; Chen, Y.; Niu, S.; Lyu, Z.; Tian, Y.; Shen, X.; Li, Y.-R.; Yang, L. Biological Functions and Therapeutic Applications of Human Mucosal-Associated Invariant T Cells. *J. Biomed. Sci.* **2025**, 32, 32.
Naïve B cells	IGHD<br>FCER2	[72] Ma, J.; Wu, Y.; Ma, L.; Yang, X.; Zhang, T.; Song, G.; Li, T.; Gao, K.; Shen, X.; Lin, J.; Chen, Y.; Liu, X.; Fu, Y.; Gu, X.; Chen, Z.; Jiang, S.; Rao, D.; Pan, J.; Zhang, S.; Zhou, J.; Huang, C.; Shi, S.; Fan, J.; Guo, G.; Zhang, X.; Gao, Q. A Blueprint for Tumor-Infiltrating B Cells across Human Cancers. *Science.* **2024**, 384, eadj4857.
	SELL	[73] Weisel, N. M.; Joachim, S. M.; Smita, S.; Callahan, D.; Elsner, R. A.; Conter, L. J.; Chikina, M.; Farber, D. L.; Weisel, F. J.; Shlomchik, M. J. Surface Phenotypes of Naive and Memory B Cells in Mouse and Human Tissues. *Nat. Immunol.* **2022**, 23, 135–145.
Memory B cells	CD27	[73] Weisel, N. M.; Joachim, S. M.; Smita, S.; Callahan, D.; Elsner, R. A.; Conter, L. J.; Chikina, M.; Farber, D. L.; Weisel, F. J.; Shlomchik, M. J. Surface Phenotypes of Naive and Memory B Cells in Mouse and Human Tissues. *Nat. Immunol.* **2022**, 23, 135–145.
	CXCR4	[74] Kashyap, M. K.; Amaya-Chanaga, C. I.; Kumar, D.; Simmons, B.; Huser, N.; Gu, Y.; Hallin, M.; Lindquist, K.; Yafawi, R.; Choi, M. Y.; Amine, A.-A.; Rassenti, L. Z.; Zhang, C.; Liu, S.-H.; Smeal, T.; Fantin, V. R.; Kipps, T. J.; Pernasetti, F.; Castro, J. E. Targeting the CXCR4 Pathway Using a Novel Anti-CXCR4 IgG1 Antibody (PF-06747143) in Chronic Lymphocytic Leukemia. *J. Hematol. Oncol.* **2017**, 10, 112.
	TCL1A	[75] Teitell, M. A. The TCL1 Family of Oncoproteins: Co-Activators of Transformation. *Nat. Rev. Cancer.* **2005**,

		5, 641–648.
	BACH2	[76] Muto, A.; Ochiai, K.; Kimura, Y.; Itoh-Nakadai, A.; Calame, K. L.; Ikebe, D.; Tashiro, S.; Igarashi, K. BacH2 Represses Plasma Cell Gene Regulatory Network in B Cells to Promote Antibody Class Switch. *EMBO J.* **2010**, 29, 4048–4061.
Plasmablasts	IRF4<br>XBP1<br>PRDM1<br>SDC1	[77] Zhao, Y.; Gong, S.; Yang, Y.; Lu, Y.; Bai, J.; Liu, M.; Bai, W.; Dong, J. CFP1 Promotes Germinal Center Affinity Maturation and Restrains Memory B Cell Differentiation through H3K4me3 Modulation. *Nat. Commun.* **2025**, 16, 8013.
	TNFRSF17<br>IGJ	[78] Phad, G. E.; Pinto, D.; Foglierini, M.; Akhmedov, M.; Rossi, R. L.; Malvicini, E.; Cassotta, A.; Fregni, C. S.; Bruno, L.; Sallusto, F.; Lanzavecchia, A. Clonal Structure, Stability and Dynamics of Human Memory B Cells and Circulating Plasmablasts. *Nat. Immunol.* **2022**, 23, 1076–1085.
Plasma cells	JCHAIN	[79] Li, Y.; Huang, H.; Wang, Q.; Zheng, X.; Zhou, Y.; Kong, X.; Huang, T.; Zhang, J.; Zhou, Y. Identification of Prognostic Risk Model Based on Plasma Cell Markers in Hepatocellular Carcinoma through Single-Cell Sequencing Analysis. *Front. Genet.* **2024**, 15, 1363197.
	MZB1	[80] Geary, B.; Sun, B.; Tilvawala, R. R.; Barasa, L.; Tsoyi, K.; Rosas, I. O.; Thompson, P. R.; Ho, I.-C. Peptidylarginine Deiminase 2 Citrullinates MZB1 and Promotes the Secretion of IgM and IgA. *Front. Immunol.* **2023**, 14, 1290585.
	DERL3	[81] Deng, F.; Jiang, W.; Wang, N.; Wu, Y.; Xu, J.; Hou, R.; Jia, F. Bioinformatics Identification of Key Genes Correlating NOD1 and Endoplasmic Reticulum Stress in Hepatitis B Virus-Induced Acute Liver Failure. *Sci. Rep.* **2025**, 15, 35919.
	XBP1	[82] Todd, D. J.; McHeyzer-Williams, L. J.; Kowal, C.; Lee, A.-H.; Volpe, B. T.; Diamond, B.; McHeyzer-Williams, M. G.; Glimcher, L. H. XBP1 Governs Late Events in Plasma Cell Differentiation and Is Not Required for Antigen-Specific Memory B Cell Development. *J. Exp. Med*. **2009**, 206, 2151–2159.
	PRDM1	[83] Cheng, R. Y.-H.; Hung, K. L.; Zhang, T.; Stoffers, C.

	SDC1	M.; Ott, A. R.; Suchland, E. R.; Camp, N. D.; Khan, I. F.; Singh, S.; Yang, Y.-J.; Rawlings, D. J.; James, R. G. Ex Vivo Engineered Human Plasma Cells Exhibit Robust Protein Secretion and Long-Term Engraftment in Vivo. *Nat. Commun.* **2022**, 13, 6110.
	IGLL5	[84] Feng, J.; Hou, Y.; Liu, C.; Wang, Y.; Chen, W.; Liu, Y.; Bian, H. IGLL5 Has Potential to Be a Prognostic Biomarker and Its Correlation with Immune Infiltrates in Breast Cancer. *Am. J. Clin. Exp. Immunol.* **2025**, 14, 111–126.
	HSP90B1	[85] B, L.; Z, L. Endoplasmic Reticulum HSP90b1 (Gp96, Grp94) Optimizes B-Cell Function via Chaperoning Integrin and TLR but Not Immunoglobulin. *Blood.* **2008**, 112.
Activated B cells	CD69<br>CD83	[86] Osmani, Z.; Beudeker, B. J. B.; Groothuismink, Z. M. A.; de Knegt, R. J.; Chung, R. T.; Aerssens, J.; Bollekens, J.; Janssen, H. L. A.; Gehring, A. J.; Lauer, G. M.; Shalek, A. K.; van de Werken, H. J. G.; Boonstra, A. B-Cell Activation Gene Signature in Blood and Liver of Hepatitis B e Antigen-Positive Patients with Immune Active Chronic Hepatitis B. *J. Infect. Dis.* **2024**, 230, e1263–e1273.
	HLA-DRA	[87] Shin, E.; Schwarz, K. B.; Jones-Brando, L. V.; Florea, L. D.; Sabunciyan, S.; Wood, L. D.; Yolken, R. H. Expression of HLA and Autoimmune Pathway Genes in Liver Biopsies of Young Subjects with Autoimmune Hepatitis Type 1. *J. Pediatr. Gastroenterol. Nutr.* **2022**, 75, 269–275.
Pathogenic B cells	LTB	[88] Tumanov, A.; Kuprash, D.; Lagarkova, M.; Grivennikov, S.; Abe, K.; Shakhov, A.; Drutskaya, L.; Stewart, C.; Chervonsky, A.; Nedospasov, S. Distinct Role of Surface Lymphotoxin Expressed by B Cells in the Organization of Secondary Lymphoid Tissues. *Immunity.* **2002**, 17, 239–250.
	TNFSF13B	[89] Novak, A. J.; Slager, S. L.; Fredericksen, Z. S.; Wang, A. H.; Manske, M. M.; Ziesmer, S.; Liebow, M.; Macon, W. R.; Dillon, S. R.; Witzig, T. E.; Cerhan, J. R.; Ansell, S. M. Genetic Variation in B-Cell-Activating Factor Is Associated with an Increased Risk of Developing B-Cell Non-Hodgkin Lymphoma. *Cancer*

		Res. **2009**, 69, 4217–4224.
	CCL3<br>CCL4	[90] Tsai, S.-C.; Lin, S.-J.; Lin, C.-J.; Chou, Y.-C.; Lin, J.-H.; Yeh, T.-H.; Chen, M.-R.; Huang, L.-M.; Lu, M.-Y.; Huang, Y.-C.; Chen, H.-Y.; Tsai, C.-H. Autocrine CCL3 and CCL4 Induced by the Oncoprotein LMP1 Promote Epstein-Barr Virus-Triggered B Cell Proliferation. *J. Virol.* **2013**, 87, 9041–9052.
	IFITM1	[91] Wilkins, C.; Woodward, J.; Lau, D. T.-Y.; Barnes, A.; Joyce, M.; McFarlane, N.; McKeating, J. A.; Tyrrell, D. L.; Gale, M. IFITM1 Is a Tight Junction Protein That Inhibits Hepatitis C Virus Entry. *Hepatology.* **2013**, 57, 461–469.
	IFI44L	[92] Zhang, K.; Luo, Z.; Yao, X.; Lu, D.; Hong, T.; Zhu, X.; Chen, M.; Wang, X. Identification of Epigenetic Alteration of the IFI44L Gene in B Cells of Sjogren's Syndrome as a Clinical Biomarker and Molecular Significance. *J. Inflammation Res.* **2025**, 18, 2499–2512.

Supplementary Table S4. The primer sequences for RT-qPCR.

Gene	Forward primer (5′ - 3′)	Reverse primer (5′ - 3′)
Kras	CAAGAGCGCCTTGACGATACA	CCAAGAGACAGGTTTCTCCATC
Dusp6	ATAGATACGCTCAGACCCGTG	ATCAGCAGAAGCCGTTCGTT
Isg15	GGTGTCCGTGACTAACTCCAT	CTGTACCACTAGCATCACTGTG
Mx1	GACCATAGGGGTCTTGACCAA	AGACTTGCTCTTTCTGAAAAGCC
Hk2	ATGATCGCCTGCTTATTCACG	CGCCTAGAAATCTCCAGAAGGG
Ldha	CAAAGACTACTGTGTAACTGCGA	TGGACTGTACTTGACAATGTTGG
Nox4	TGCCTGCTCATTTGGCTGT	CCGGCACATAGGTAAAAGGATG
Sod2	CAGACCTGCCTTACGACTATGG	CTCGGTGGCGTTGAGATTGTT
Notch1	GATGGCCTCAATGGGTACAAG	TCGTTGTTGTTGATGTCACAGT
Hes1	TCAACACGACACCGGACAAAC	ATGCCGGGAGCTATCTTTCTT
Mtor	CAGTTCGCCAGTGGACTGAAG	GCTGGTCATAGAAGCGAGTAGAC
Rps6kb1	GGGGCTATGGAAAGGTTTTTCA	CGTGTCCTTAGCATTCCTCACT
